# Crops Change the Morphology, Abundance, and Mass of Microplastics in Mollisols of Northeast China

**DOI:** 10.3389/fmicb.2022.733804

**Published:** 2022-04-04

**Authors:** Jiuqi Wang, Pengke Yan, Wan Wang, Xinhua Hao, Bing Xu, Muhammad Aurangzeib, Shaoliang Zhang

**Affiliations:** Northeast Agricultural University, Harbin, China

**Keywords:** maize, soybean, straw, enzymes, plastics, black soil

## Abstract

Degradation of microplastics (MPs) by both physicochemical and biological processes in the natural environment is determined by the enzymes inside the soil, and which was severely influenced by crop growth and straw amendment (SA). However, it is still unclear how crop growth and SA influence degradation of MPs in soils. In this study, both catalase and sucrase were measured, and the stereomicroscope combined with microscopic infrared spectroscopy and scanning electron microscope (SEM) was used to detect the morphology and quantity of low-density polyethylene microplastic (LDPE-MP) and low-density polypropylene microplastic (LDPP-MP), after crop growth (maize and soybean, with and without SA, 1 and 2% MP) in an outdoor pot experiment, in the Mollisols. The results showed that the growth of the crops changed the morphology, functional groups (e.g., methylene, carbonyl), total mass, and abundance ratio of MPs of different sizes. These were possibly caused by enzymes that were significantly influenced by crop types, abundance, and types of MPs in the soils. Maize growth decreased the mass of LDPE-MP and LDPP-MP by 28.7 and 32.7%, respectively, and 2% (w/w) of LDPP-MP addition in soil decreased mass of 9%, which was higher than that in 1% (w/w) LDPP-MP addition in soil. Soybean growth with SA decreased the mass of LDPE-MP and LDPP-MP by 36.6 and 20.7%, respectively, than the control treatment (CK). Compared with CK, both crop growth and SA changed the abundance of MPs of different sizes and decreased the mean size of MPs. The LDPE-MP could be more easily degraded by enzymes in the soils compared to LDPP-MP when the MP size was smaller with surface roughness. Generally, both maize and soybean growth can accelerate MP change in soils, and MP change process was mainly determined by SA, MP types, and the dose effect of MP.

## Introduction

The advantages of using plastics are relative stability, low cost, low density, and nontoxicity, hence, they have been widely used in the industry, agriculture, and daily use (Thompson et al., 2009; Plastics Europe, 2019; Zhang et al., 2020a; Statista, 2021). Researchers reported that plastic production has increased every year since the 1950s, and even exceeded 83 Mt in 2017. Worldwide, the production is expected to reach 590 million Mt in 2050, whereas recycling was less than 9% in the current scenario (Thompson et al., 2009; Zhang et al., 2020a; Statista, 2021). Some publications indicate that plastics, especially microplastics (MPs, particle size <5 mm) from plastics degradation, can cause adverse effects on biota in both the terrestrial and aquatic ecosystems worldwide (Zhang et al., 2019b, 2021). Thus, plastic pollution, especially those related to the critical level of MP, is regarded as one of the most vital environmental issues raised in recent decades (Zhang et al., 2019b, 2021; Wang et al., 2022). Since the terrestrial system is a significant source of MP for oceans, recently, several researchers have focused on the degradation, migration, and distribution of MP in soils (Zhang et al., 2019a, 2020a; Wang et al., 2021).

It is worth mentioning that the MP degradation process influences the dynamics of MP size, morphology, and distribution in soils. However, there are still fewer reports available on the key factors influencing MPs degradation in soils (Zhang et al., 2020a). Generally, the bigger and coarser the specific surface area of the MP is, the greater their influence by microorganisms, enzymes, and external environment is, especially the pits and flakes were easily colonized by various microorganisms (Besseling et al., 2017; Zhang et al., 2019b). Degradation of MPs could be caused by certain enzymatic activities and lead to polymer chain cleavage, resulting in the formation of oligomers and monomers (Montazer et al., 2018a,b). Previous studies have indicated that foreign material, which is organic and beneficial, especially the straw amendment (SA), tends to improve the activities of soil microorganisms and increases the training and richness of enzymes in soils (Zhang et al., 2019b). It is worth mentioning that soil enzymes are released by the microorganisms, and plant roots even account for 90% of soil enzymes (Lynch and Whipps, 1990). Plants perform key roles that influence the ecological process in the terrestrial systems and control the transformation of substances in soils (Zhuang et al., 2013). However, it is still unclear how crop growth influences soil degradation. Zhang et al. (2020a) found the low-density polyethylene microplastics (LDPE-MPs) in surface and deep soil layers in the farmland, which were positively correlated to macroplastics (MAPs) on a large scale but were not correlated to MAPs on a small scale. They deduced that LDPE-MP distribution was not only influenced by water movement but also possibly by enzymes (Zhang et al., 2020a). Thus, both crop growth and SA alter the soil ecosystems, further combining with microorganisms to change the activity of the enzymes and possibly affect the degradation of MPs in soils.

Plastic film and changed mulch mainly comprise low-density polyethylene (LDPE) and low-density polypropylene (LDPP), which cause severe pollution and damage the soils when they are not recycled and degraded into MP debris in soils for a long time (Zhang et al., 2021). Mollisols are mainly distributed in the high-cold areas in the world, which have high soil organic matter (SOM) as well. In these areas, the agricultural film has been widely used to increase soil moisture and temperature with a low recycling rate for a long time, which seriously results in both plastic and MP pollution. In this study, the influences of maize and soybean growth, and SA on the morphology, surface functional groups, abundance, and mass of MPs with different sizes were tested. The changes in enzymes activity in response to crop growth, microorganism, and MP degradation were also measured after 5 months. The primary purposes of this study are to validate the following hypotheses: (1) MP degradation is influenced by both crop growth and SA and could be correlated to the activity of enzymes in soils; and (2) MP degradation was also influenced by crop types and MP composition. The results are beneficial to understand the MP dynamics and driving mechanism more deeply in soils and provide guidance for a better regulation of MP pollution in farmland.

## Materials and Methods

### Soils and Microplastics

A pot experiment was used in this study, and soils were taken from the farmland of the Harbin region in Heilongjiang Province of Northeast China (no history of plastics application). The soil was classified as typical black soil according to the Chinese Soil Classification (CST) and Mollisols in the United States Soil Classification (USST) (Zhang et al., 2017). The basic physicochemical properties of soil are shown in Table 1. The soils were mixed thoroughly, air-dried at room temperature, and sieved at 2 mm before the experiment. No MP components were detected in these soils using a Fourier transform infrared spectroscopy (FTIR) (Supplementary Figure 1).

**TABLE 1:** Physicochemical properties of the soils used in the experiment.

pH	SOM (g/kg)	Total nitrogen (g/kg)	Available nitrogen (mg/kg)	Total phosphorus (g/kg)	Available phosphorus (mg/kg)	Total potassium (g/kg)	Available potassium (mg/kg)
7.7	23.6	2.95	133.53	0.87	68.66	25.1	186

Because the LDPE and LDPP have been widely used in the farmland of the world and were broadly used in Heilongjiang Province (Zhang et al., 2018, 2020b), LDPE-MP and low-density polypropylene microplastic (LDPP-MP) were adopted in this experiment. The irregular shape of PE and PP was changed into the size of 100 mesh particles, which were ground by the same manufacturer in the same production mode and were dried and screened before leaving the factory (both were produced by Red Star Plastics Co., Ltd., Anqing City, China). In addition, the specific LDPE-MP and LDPP-MP were also measured by a sieving method before the experiment. In order to quantify the degradation, the characteristics of the original microplastic were measured and defined as treatment “original.” LDPE-MP and LDPP-MP were white in color having sizes of <2 mm (Table 2).

**TABLE 2:** Density, particle sizes, and morphology of polyethylene (PE) and polypropylene (PP) used in this study.

	Density (g cm^–3^)	Color	Shape	w/w, %				
Particle size	–			75–125 μm	125–150 μm	150–300 μm	300–500 μm	500–2,000 μm
PE	0.92	White	Spherical, fiber	7%	20%	25%	15%	33%
PP	0.91	White	Spherical, fiber	3%	20%	25%	19%	33%

### Experimental Design, Management, and Soil Sampling

**FIGURE 1:**
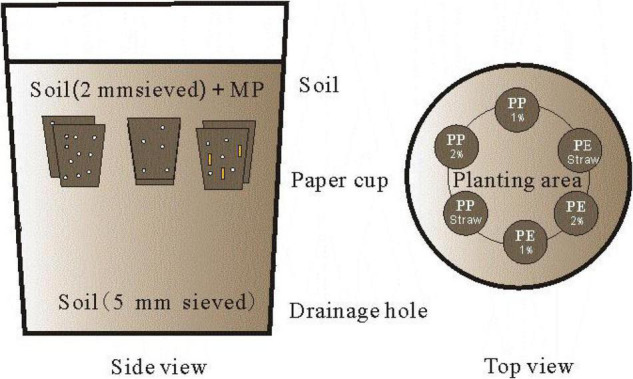
The side view and top view of the incubation device were designed. Microplastics and maize straw were added into paper cups and then buried at 20 cm soil depth in the bucket for incubation. The soil outside the paper cup should be sieved 5 mm to distinguish it clearly from the soil inside the paper cup.

A compound method of paper cups combining pot was adopted to study the crop growth and SA on MP degradation, and this method can effectively reduce the MP pollution (reducing the MP dose) in the experiment (Figure 1). Then, 100 ml of six paper cups combined with a PVC bucket of 20 L was used in each pot in the incubation experiment. The bottoms of the cups were pre-punched, and multilayer filter papers (<4 μm) were preset on the bottom to ensure water infiltrating while avoiding the MP leaching (the device had been tested repeatedly three times). PVC buckets were pre-perforated in the bottom and were cleaned before the experiment.

For the treatments of 1% PE, 2% PE, 1% PP, and 2% PP, each paper cup was filled with a homogeneous mixture of 100 g of dry soils (sieved at 2 mm) and MP (LDPE-MP or LDPP-MP). In addition, in treatments of 1% PE + SA, 1% PP + SA, each paper cup was filled with a homogeneous mixture of 100 g of dry soils (sieved at 2 mm), MP (LDPE-MP or LDPP-MP), and SAs (maize straw). The content of MP addition in the paper cup was 1 (w/w) and 2%, and the range of SA was 0.5% (w/w). Each bucket was filled with 1 kg of dry soil (sieved at 2 mm) at the beginning, and then six paper cups with a mixture of soils were evenly arranged on the surface of the earth (Figure 1). The gaps between the paper cups were filled with the soil (sieved at 2 mm), and then the soils were continuously added until the bucket was full. All paper cups were buried at 20 cm soil depth in each bucket.

Soybean (*Glycine max*, Dong Nong 254th) and maize (*Zea mays*, Dong Nong 265th) were most popularly planted in the Mollisol of Northeast China and were used in this experiment. Three soybean seeds were planted at each point, and five seeding points were evenly distributed around the surface of soils in the bucket. In addition, three seeds of maize were planted in the center of the surface of soils in the bucket. Only one plant of soybean/corn was allowed to remain in the buckets when the height of soybean and maize was 5 cm. No crop was planted in the control treatment (CK). Each treatment had four replicates, and all buckets were randomly arranged in an area of the experimental station. Depending on the weather condition, all treatments were watered with the same volume of distilled water once in 2–4 days. Plastic pollution was avoided throughout the experimental preparation, management, and soil sampling.

The average total rainfall was 570.7 mm from June to August in Harbin of 2019. Seeds were sown in May, and crop harvesting and soil sampling were all carried out in October after one growth cycle of the crop. During the soil sampling process, firstly, surface soils (0–20 cm) were removed; secondly, the root system around the paper cups was cut off, and then paper cups were collected. Crop roots were picked out directly from soils for each paper cup, and then soil samples were mixed evenly, and half of the soils were dried for MPs analysis, while another half of soils was frozen for enzymes analysis.

### Separation and Determination of Soil Microplastics

To avoid plastic pollution during the separation and determination of soil MPs, we cleaned the laboratory thoroughly before the experiment and kept it clean during the entire test. In addition, during the experiment, it was not allowed to wear clothes and hats made of plastic fibers. The separation of MPs involved two steps, and it has been proved that the extraction rate (MP size (50 (m) is well over 95% (Zhang et al., 2018):

(1) A total of 10 g of the soil sample was weighed and added into a glass beaker (100 ml). Then, 60 ml of sterile distilled water was added and stirred with a glass rod, then 40 ml of sterile distilled water was used to clean the MPs from glass rod to beaker. The breaker was kept static over 24 h until the suspension appeared and all high-density substances sunk. MPs were floated onto the surface and were filtrated by the fine filter papers (pore diameter (3 (m). Then, the filter papers with LDPE-MPs and LDPP-MPs were oven dried to the consistent weight at 60°C.
(2) LDPE-MPs and LDPP-MPs were transferred into beakers and were processed by Fenton’s reagent (20 min, 25°C, pH (4) to remove impurities that mainly included organic matter (Hurley et al., 2018), then dried at 60°C (consistent weight) again and weighed by analytical balance (FA/JA, Shanghai Yueping Instrument Co., Ltd., precision of 0.00001 g). For avoiding the pollution caused by foreign impurities during the experiment, tin lids were used to cover all beakers.

The abundance of LDPE-MPs and LDPP-MPs with different sizes was calculated by a high-resolution camera (IS Capture 3.7.8, Tucsen Photonics Co., Ltd., China) connected with a stereomicroscope (×50, Novel NSZ-608T) and ImageJ software (Zhang et al., 2018). After 5 months, the surface morphology of LDPE-MP and LDPP-MP for treatments of crops growth and SA was measured by a scanning electron microscope (SEM, Hitachi, S3400). The FTIR spectroscopy (Nicolet iN10, Thermo Scientific) was used to investigate the structure and functional groups of LDPE-MP and LDPP-MP. For each mid-infrared (mid-IR) spectrum (4,000–750 cm^–1^), 16 scans were collected in reflection mode at 4 s^–1^ frequencies. The background was a pure golden mirror. The IR spectra of LDPE-MP and LDPP-MP were determined by carbonyl index (CI) to measure the formation of several by-products containing carbonyl functionalities on the surface of the MPs that result from the oxidation of the material (Barbes et al., 2014; Andrady, 2017). CI is the ratio of the carbonyl peak and reference peak (Almond et al., 2020). In this study, CI is calculated by the following formula:


$$\mathrm{Carbonyl}\,\text{Index}={\text{A}}_{C=O}/{\text{A}}_{-\mathrm{CH2}}$$

where A_c=o_ is the absorbance of the carbonyl peak, and A_–CH_ is the absorbance of the reference peak. Therefore, the CI was used in this work as a metric for the oxidation of polymers, enabling the quantification of some of the changes in the chemical structure of the material.

### Measurement of Enzyme and Straw Degradation Rate

Oxidoreductases (oxidases and dehydrogenases) and hydrolases have been found to significantly promote the changes in the morphology and mass of MPs in soils, especially on LDPE degradation (Qi et al., 2018). The activities of catalase (typical oxidases) and sucrase (specific hydrolases) were measured in this experiment. Sucrase and catalase activities were determined by the 3,5-dinitrosalicylic acid method and the hydrogen peroxide consumption method. Debris of straw was picked out under a magnifying glass from the soils after air-drying and weighed by an analytical balance (FA/JA, Shanghai Yueping Instrument Co., Ltd., precision of 0.00001 g).

### Statistical Analysis

The data are presented as the mean ± SD in this study. Significant differences (*p* < 0.05) were tested *via* two-way ANOVA. The significant difference between treatments was conducted by least significant difference (LSD) tests (*p* < 0.05). LSD tests were conducted to compare the significant differences among different crop growth on the same incubation day (*p* < 0.05). LSD tests were also used to compare the difference between with and without SA in the same periods (*p* < 0.05). All figures were drawn using Origin 2019. Statistical analyses were carried out using SPSS 22.0. Spectra used for analysis and making figures were the average of the spectra of four random particles and were analyzed using OMNIC 8.2 (Thermo Scientific).

## Results

### Crop Growth Changed the Morphology and Function Groups of the Surface of Microplastics in the Soils

Original LDPE-MPs were composed of various sizes of particles that were typically connected. After crop growth at 5 months, LDPE-MP had a smaller size, sharper edge, and rougher surface compared with the original LDPE-MP particles (Supplementary Figure 2A). Similarly, LDPP-MP sizes were also obviously reduced after crop growth at 5 months compared to the original LDPP-MPs, and the surface became rough and even formed many cavities on the surface of MP (Supplementary Figure 2B). The differences between treatments with and without SA were also noticeable, and the morphological changes were more evident with SA than without.

**FIGURE 2:**
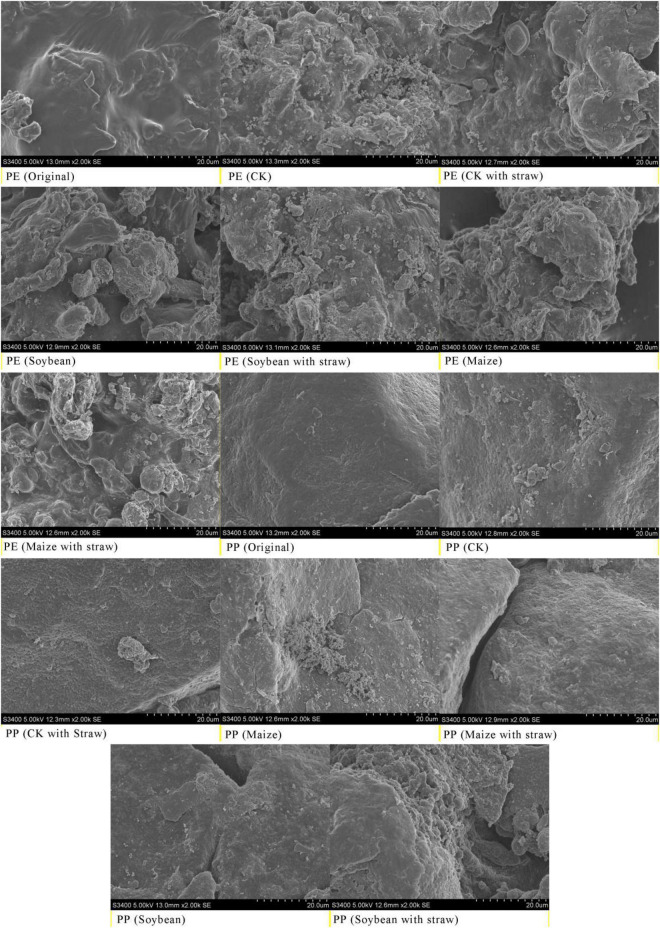
Surface morphology of low-density polyethylene (LDPE) and low-density polypropylene (LDPP) was changed before and after soybean (*Glycine max*) growth, maize (*Zea mays*) growth, and straw amendment. Images were taken by a scanning electron microscope (SEM, Hitachi, S3400). CK was the control treatment with no crop growth; the original was LDPE-MPs and LDPP-MPs without any treatment. Here, only treatments of 1% LDPE and LDPP are shown.

The original particles of LDPE-MPs and LDPP-MPs had a smooth surface, especially for LDPP-MP (Figure 2), while they were coarser with an irregular shape and a rough surface after growth at 5 months. Primarily there was more difference on LDPE-MP surface when treated with SA (Figure 2).

**FIGURE 3:**
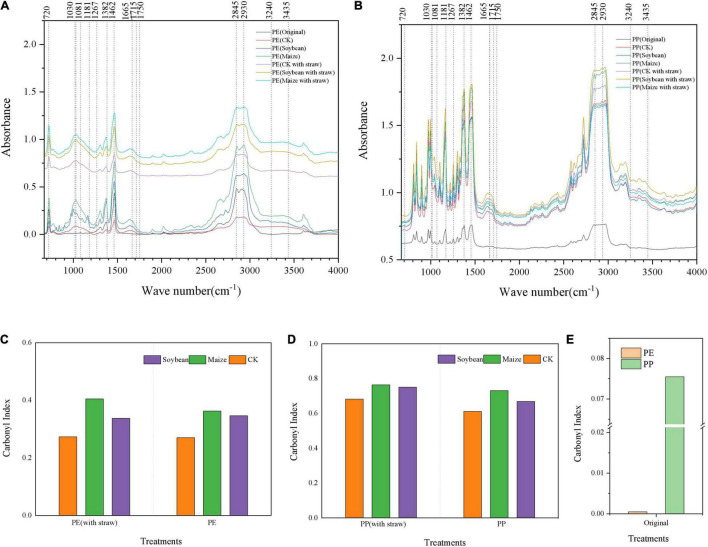
The Fourier infrared spectra and carbonyl index of low-density polyethylene (LDPE) and low-density polypropylene (LDPP) microplastics were different in the treatments of crops growth (soybean and maize, with and within straw addition). **(A)** LDPE-MP and **(B)** LDPP-MP. **(C)** Carbonyl index of LDPE-MP, **(D)** carbonyl index of LDPP-MP, and **(E)** carbonyl index of original LDPE-MPS and LDPP-MPS. CK was the control treatment without crop growth, and the original was LDPE-MPS and LDPP-MPs before the experiment. All spectra were the average spectra of the spectra of four random particles and were analyzed by microscopic infrared spectroscopy (Nicolet iN10, Thermo Scientific) using OMNIC software (Thermo Scientific).

With the crop growth, the new peaks appeared in the spectrograms of LDPE-MP, which were initially not found in the original LDPE-MP, such as signals were found near 1,030, 1,665, and 3,435 cm^–1^ (Figure 3), while no obvious new peaks appeared in LDPP-MP. Compared with the original LDPP-MP, after 1 year of crop growth, the peak area near 1,665 cm^–1^ increased compared with the peak area of methylene (1,490–1,420 cm^–1^). Moreover, the peak value near 1,780–1,600 cm^–1^ represents carbonyl, which was changed in all treatments and had the larger area in treatments with crop growth and SA (compared with methylene, 1,490–1,420 cm^–1^). CI was higher in treatments of crop growth than those in CK and was significantly greater in the maize treatment compared to soybean treatment (*p* < 0.05). Additionally, the CI was higher in treatments of SA than those in the treatments without SA (Figures 3C–E).

### Crop Growth Changed the Abundance of Microplastics in the Soils

**FIGURE 4:**
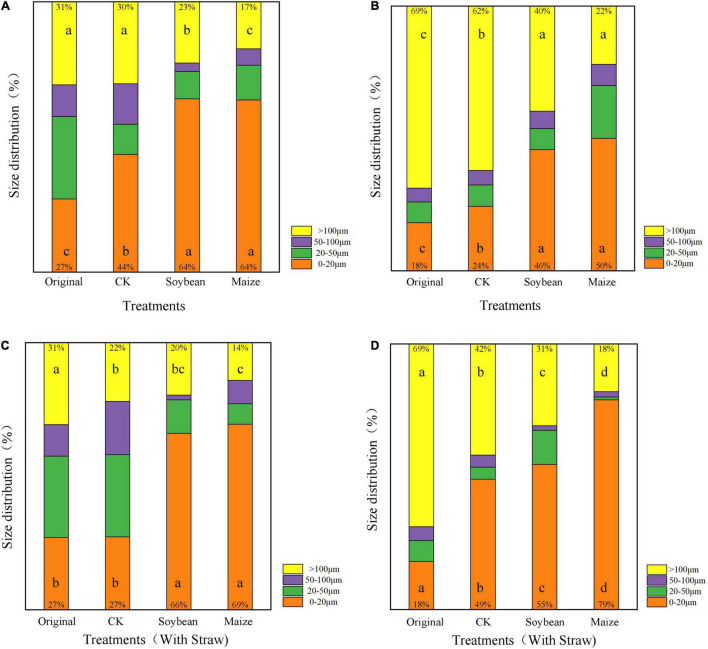
The abundance proportion of single-particle size to the total particle size of low-density polyethylene (LDPE) and low-density polypropylene (LDPP) microplastics in the treatments of crop growth (soybean and maize, with and without straw addition). **(A)** LDPE-MP, **(B)** LDPP-MP, **(C)** LDPE-MP (with straw), and **(D)** LDPP-MP (with straw). CK was the control treatment without crop growth, and the original was LDPE-MPs and LDPP-MPs without any treatment. Columns followed by the same letter at the same particle size were not significantly different based on a least significant difference (LSD) multiple range test (*p* < 0.05).

Compared with the original LDPE-MP and LDPP-MP, the abundance composition of MP particle sizes was significantly changed in CK treatments (Figure 4) (*p* < 0.05). Compared with the original LDPE-MP, the abundance of LDPE-MP size >100 μm decreased by 16.1%, while the abundance of size <20 μm increased by 37.0% in CK treatments (Figure 4A). Similarly, compared with the original LDPP-MP, the abundance of LDPP-MP size >100 μm was reduced by 10.1%, while the abundance of LDPP-MP size <20 μm increased by 33.3% in CK treatments (Figure 4C). The change of abundance composition of MP sizes in CK treatments was more obvious in LDPE-MP than in LDPP-MP.

Compared with CK, the abundance of LDPE-MP size >100 μm decreased in all treatments, while the size <20 μm increased. Crop growth significantly altered the abundance composition of the LDPE-MP and LDPP-MP with different sizes (*p* < 0.05). Compared with CK, the abundance of LDPE-MP with size >100 μm decreased by 23.3% in maize treatments, while the size of <20 μm increased by 73.0% (Figure 4A). Similarly, compared with CK, the abundance of LDPP-MP with the size of >100 μm decreased by 43.3%, while the size of <20 μm increased by 91.7% in treatments with maize growth (Figure 4B).

Compared with CK, the abundance of LDPE-MP with size >100 μm was less than 13.5%, and the abundance of LDPP-MP with <20 μm was 104.2% greater in the treatments with SA (Figures 4C,D). The change of abundance composition of LDPE-MP and LDPP-MP sizes between treatments with SA and CK was more obvious in maize treatments than in treatments with soybean growth. The abundance of LDPE-MP sizes >100 μm decreased by 36.4 and 17.6% in the treatment of maize growth with SA compared to CK and maize treatment without SA, while <20 μm increased by 155.6 and 7.8%. Compared with the CK and maize treatment without SA, the abundance of LDPP-MP size >100 μm decreased by 57.1 and 18.2% in maize treatment without SA, while <20 μm increased by 61.2 and 58.0%.

### Crop Growth Changed the Mass of Microplastic in the Soils

Microplastic mass was reduced in all treatments after crop growth at 5 months, and the mass of MP was significantly (*p* < 0.05) lower in treatments of maize and soybean growth than that in CK. Compared with CK, LDPE-MP mass was decreased by 28.7% in maize treatments while the LDPP-MP mass was decreased by 32.7%. The change of MP mass was typically lower in LDPP-MP treatments than that in treatments of LDPE-MP addition.

**FIGURE 5:**
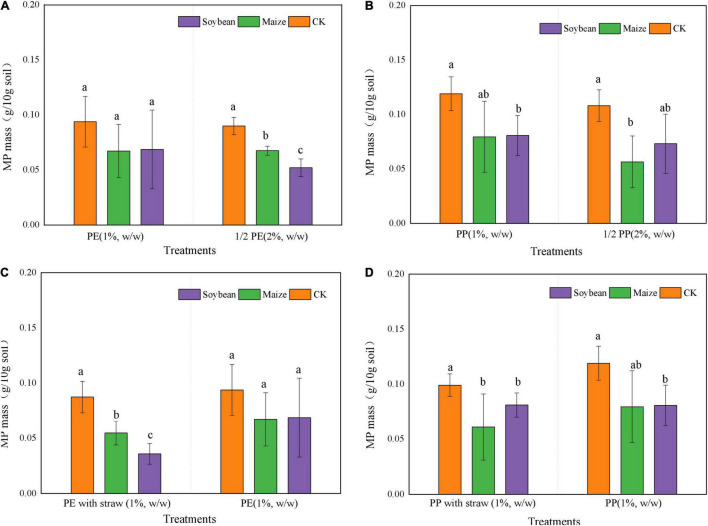
Mass content (g 10 g^– 1^ soil) of low-density polyethylene (LDPE) and low-density polypropylene (LDPP) differed in the treatments of crop growth (soybean and maize, with and within straw addition). **(A–D)** LDPE-MP, LDPP-MP, LDPE-MP (with straw), and LDPP-MP (with straw). CK was the control treatment without crop growth. Columns followed by the same letter were not significantly different based on a least significant difference (LSD) multiple range test (*p* < 0.05).

The mass reductions were higher in 2% LDPP-MP and LDPE-MP treatments than that in the treatments of 1% MP addition with crops growth and CK (Figures 5A,B). In order to compare with 1% MP addition, the y-axis of MP mass value was shrunk to the proportion of half for 2% LDPP-MP and LDPE-MP addition in Figure 5. When treatments experienced the soybean growth, MP mass in treatment of 2% LDPE-MP addition was lower than that in 1% MP addition but was not significant. Moreover, MP mass was 9% lower in 2% LDPP-MP addition than that in 1% LDPP-MP addition (*p* < 0.05). At the same time, compared with the treatments of maize growth without SA, the mass of soil LDPE-MP with soybean growth was reduced by 36.6% in the treatments with SA when 1% LDPE-MP was added (Figures 5C,D). Compared with the treatments with soybean growth without SA, the LDPP-MP mass decreased in treatments of maize growth and SA even increased by 20.68% with SA when 1% LDPE-MP was added.

### Soil Enzyme Activity and Straw Degradation

**FIGURE 6:**
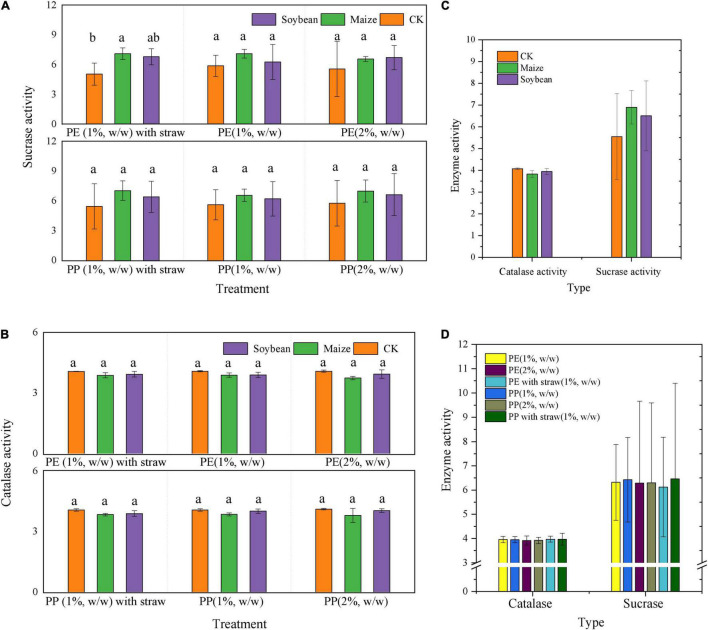
Enzyme activity of sucrase and catalase differed in the soybean and maize growth treatments, PE, PP addition, and with and without straw addition. **(A)** Sucrase, **(B)** catalase, and **(C)** enzyme activity with different crop growth. **(D)** Enzyme activity with different contents and types of microplastics and straw addition. CK was the control treatment without crop growth. Columns followed by the same letter were not significantly different based on a least significant difference (LSD) multiple range test (*p* < 0.05).

**FIGURE 7:**
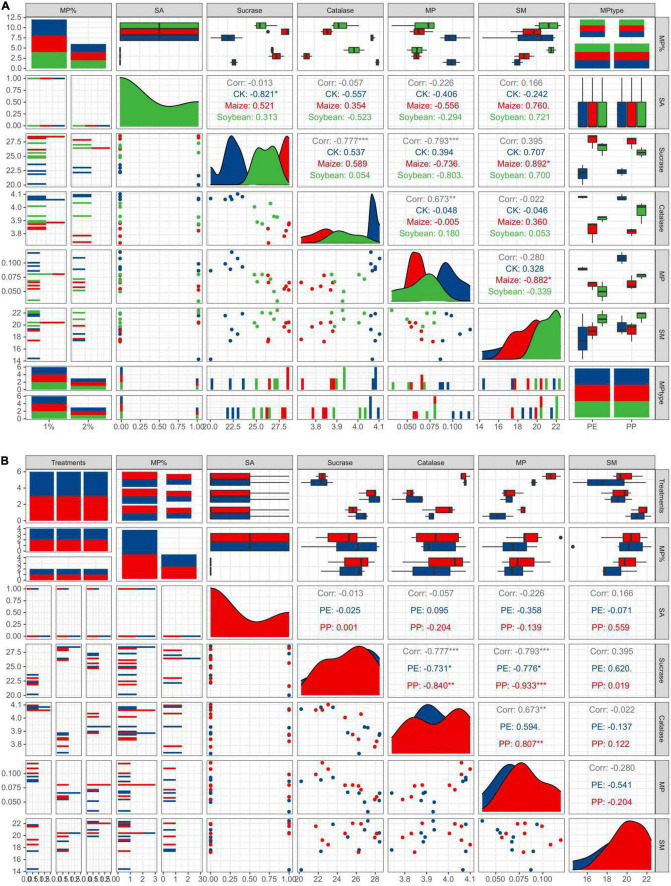
Easy pairs with different crop treatments and different MP types. **(A)** A correlation analysis between indicators in different crop treatments. **(B)** A correlation analysis between indicators under different MP types. MP% is the original MP addition. MP is MP mass after crop growth.

**FIGURE 8:**
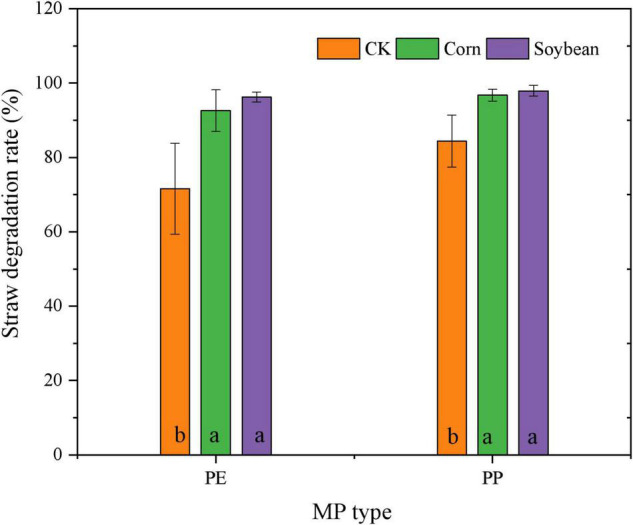
Mass content (%) of straw degraded in the treatments of crop growth (soybean and maize, different types of MP). Columns followed by the same letter were not significantly different based on a least significant difference (LSD) multiple range test (*p* < 0.05).

Sucrase and catalase activity in LDPE-MP addition treatments was slightly higher than that in PP treatments (Figure 6). However, sucrase activity was also slightly higher in crop treatments and was significantly higher in maize treatment than that in no crop treatment (CK) (*p* < 0.05) when SA was used (Figure 6A). However, sucrase activity was significantly negatively correlated with PE and PP mass (*p* < 0.05) in crop treatments, while it was not significant in CK (Figure 7). Catalase activity was significantly and positively correlated with PP mass (*p* < 0.05) in CK, while it was not correlated with MP mass in all crop treatments (Figure 7). Thus, out of sucrase and catalase, in our study, only sucrase may play a major role in MP degradation, which was influenced by crop. Interestingly, straw degradation was significantly higher in soybean treatments than in maize treatments (*p* < 0.05) (Figure 8).

## Discussion

Microplastics were constantly broken and degraded by physicochemical and biological processes and were finally fragmented into small pieces with various shapes and sizes (Zhang et al., 2019a). MPs promoted colonization, biofilm production, and the transfer of environmental microorganisms (Wang et al., 2021). Plastic degradation was mainly determined by their molecular structure in soils and typically reflected by morphology or change in mass (Ng et al., 2018; Wang et al., 2021). As reported before, the complete mineralization of PE may take hundreds or even thousands of years, while the total mineralization of PP may take 20–30 years (Zhang et al., 2019b). It is worth mentioning that MP change caused by weathering processes is slow and mainly happens on the surface of soils, while MP changes driven by biological processes are quicker in the deep soil layers (Veerasingam et al., 2016). In this study, MPs were buried at 20 cm soil depth, which could not be changed by photochemical reaction, while being mainly influenced by biological processes.

### Microplastics Change in Soils After One Growth Season

Previous reports indicated that the reduced rate of PE mass reached 0.27 g day^–1^ under specific microorganisms (Hu et al., 2020; Zhang et al., 2021), such as *Actinobacteria*, *Bacteroidetes*, *Proteobacteria*, *Cupriavidus*, and *Pseudomonas*, which significantly changed the morphology, quantity, and mass of MPs in soils, especially *Micrococcus*-Cu. Necator H16 even decomposes 1.5% LDPE-MP per day (Montazer et al., 2018a). It is worth mentioning that microorganisms promote the degradation and change the morphology, mass, and abundance of MP in soils by producing the enzymes such as oxidoreductases (oxidases and dehydrogenases) and hydrolases (Qi et al., 2018; Zhang et al., 2021). Furthermore, some reports indicated that roots could also produce enzymes during the growth process, which takes up 90% of soil enzymes (Zhang et al., 2020b). Research has shown that MPs may stick to the roots, affect the absorption of water and nutrients, and cause roots to secrete specific enzymes to degrade them (Wang et al., 2019). In addition, root exudates can increase phosphatase, invertase, and catalase, thus advancing the microbial biomass and fungal abundance in the soils (Noman et al., 2021). In this study, both LDPE-MP and LDPP-MP mass were significantly reduced by 12.1–32.7% of original addition in soils after a growth season of 5 months (Figure 5), and the morphology of LDPE-MP and LDPP-MP was changed (Figure 2 and Supplementary Figure 2). Because the enzymes of sucrase and catalase are highly related to degradation of MPs (Qi et al., 2018), these were also detected in this study. In this study, sucrase (belonging to specific hydrolases) may play a major role that was extremely significantly and positively correlated with MP mass (*p* < 0.01). In addition, functional groups of carbonyl and hydroxyl appeared or increased on the surface of MPs (LDPE-MP and LDPP-PP) in all treatments after crop growth (Figure 3), and these functional groups made the MPs more easily biodegraded and released more small MPs in soils (Yang et al., 2018). Thus, MP was possibly also degraded by enzymes produced by microorganisms or crop roots in soils. The specific surface of MPs was also more easily colonized by microorganisms forming the biofilm, which can further accelerate the morphological alterations and biodegradation of MPs (Wang et al., 2021). Compared with the treatment without growth, the CI of both LDPE-MP and LDPP-MP increased after 1 year of crop growth (Figures 3C–E), indicating that crop growth could also accelerate both LDPE-MP and LDPP-PP degradation.

### Microplastic Change Influenced by Microplastic Types

Interestingly, in this study, the mass and abundance of MP decrement were higher in treatments with LDPE addition than the treatments of LDPP addition in the same MP dose addition, and the change of LDPE-MP morphology was more obvious compared with LDPP-MP (Figures 2, 5). Because the methyl side branch of PP is easily broken by the biochemical process, the degradation period of PE is typically longer than PP (Danso et al., 2019; Zhang et al., 2021). In our study, a few hydroxyl and carbonyl functional groups were found in the original LDPP-MP, which means that LDPP-MP was more easily degraded than LDPE-MP. While the sucrase activity was significantly and positively correlated with both PP and PE mass, the gradient of LDPP mass vs. sucrase activity is higher than that for LDPE mass vs. sucrase, which means that LDPP was more susceptible to enzymes (Figure 7 and Table 3). Previous studies indicated that the structure and properties of MPs are the main factors that determine the degradation degree of MPs, and the degradation rate typically increased with the surface area due to the microorganisms being more easily colonized on a rough surface, especially for the pits (Besseling et al., 2017; Zhang et al., 2019a). In our study, the materials of LDPE-MP had a smaller size and higher roughness, and the surface area was higher than LDPP-MP when the mass addition was the same. Thus, LDPE-MP was easily degraded compared with LDPP-MP in soil. In addition, the original LDPE-MP size was smaller than LDPP and could be easily absorbed by crops. Therefore, both degradation and absorption could be the reason that the LDPE-MP decrement was higher than LDPP-MP in this study.

**TABLE 3:** Polyethylene (PE) and polypropylene (PP) mass in this study.

MP type	Enzyme type	*a*	*b*
PE	Sucrase	–0.006774	0.2696
	Catalase	0.04180	–0.06714
PP	Sucrase	–0.007191	0.3047
	Catalase	0.2387	–0.8206

*y is MP mass, x is enzyme activity, y (a × x (b, a and b are in table.*

### Microplastic Change Influenced by Crop Growth

Our study also found that the abundance and mass of MP decrement were generally higher in the maize-planted treatments than in the soybean treatments. The surface topography and functional groups (especially CI) changes in MP in maize treatments were more evident than those in the treatments of soybean. Maize has massive fibrous roots and can grow fully in the PVC bucket in our research, while soybean has a taproot system whose distribution is not tight. This study also found that the root weight was higher in the paper cups of maize treatment than that in the soybean treatment (Supplementary Figure 3). A previous publication indicated that roots could absorb MP, and MPs can further be transported into both stems and leaves (Li et al., 2019). In addition, the transpiration efficiency of C4 plants (such as maize) is higher than that of C3 plants (such as soybean) (Gao et al., 2009). In addition, compared with soybean, the enzyme types in soils of maize treatment were relatively higher (Yang and Wang, 2002), and maize produces more quantity of total carbon and total nitrogen, which are beneficial to increase the activity of microorganisms (Yin, 2009). Interestingly, sucrase activity was highest in maize growth treatments, followed by soybean growth and CK, and was significantly negatively correlated with MP mass in all crop treatments but was not negatively correlated with MP mass in treatments without crop growth. This may hint that sucrase can effectively degrade both LDPE and LDPP in soils of the crop growth treatment, and sucrase activity was mainly determined by crop growth, especially in the maize growth (Figure 7). However, catalase activity showed no difference between treatments with and without crops, while catalase activity significantly increased with LDPP increasing mass (*p* < 0.05) but was not negatively correlated with MP mass in treatment with crop growth. This may be hinting that catalase was not mainly influenced by crops, while it was mainly produced by the special microorganisms that were preferable to colonize on the LDPP surface. Generally, in this study, maize has a more vital ability to change MP than soybean planted, possibly mainly influenced by both absorption and enzymes.

### Microplastic Change Influenced by Straw Amendment

It is worth mentioning that SA applications can also accelerate the decrement of MP mass and abundance. The morphology and functional groups (especially CI) changes in MP with the SAs were also more obvious (Figures 2, 4). A previous study reported that maize stalks amendment significantly increased the abundance of *Actinobacteria*, *Ascomycota*, and *Mortierellomycota* and significantly increased the activity of microorganisms (Ocio et al., 1991). In addition, in this study, sucrase activity was significantly higher in maize treatment than that in CK (*p* < 0.05) when SA was used, but no obvious differences in treatments have been observed without SA (Figure 6A). Thus, we deduced that SA accelerated the MP degradation, possibly because of changing microecosystems in soils.

### Microplastic Change Influenced by Dose Effect

In this study, MP mass reduction percentage was higher in 2% of MP content than 1% of MP content in the same crop treatments. Other publications also found that increasing LDPE-MP addition significantly increased soil microbial respiration and urease and phosphatase enzyme activities (Qi et al., 2018). In this study, catalase and sucrase activity of 2% of MP content treatment was slightly higher. Thus, increasing the doses of LDPE-MPs and LDPP-MPs may provoke soil self-repair mechanisms and accelerate the degradation of MP mass because MP was also the carbon source in the soils.

Microplastic mass decrement was higher in this study than that in other publications, which may be due to the (1) particle size <3 μm leach out of the filter paper (φ < 3 μm) was not detected; (2) MP particles with the acceptable size, especially for the nanometer size in soils were adsorbed by crops (Li et al., 2019; Zhang et al., 2021); (3) the Fenton’s reagent corroded the parts of MP particles during the analysis process of OM degradation; and (4) the enzymes produced by both microorganisms and roots accelerated the MP degradation. It is worth mentioning that the changes in MP in the morphology, size, quantity, and mass may cause adverse effects on the ecological environment. In addition, the smaller the size of MP is, the larger the harm of MP is to the ecosystems (Zhang et al., 2021). Therefore, crop growth and SA accelerate MP change, increase MP debris, increase MP migration, and further cause more negative effects on biota, even threatening the aquatic organism when they continuously enter the aquatic ecosystems.

## Conclusion

Both maize and soybean growth can accelerate the changes in the morphology, abundance, and mass of MPs in soils. Maize growth has a more vital ability to reduce MP in soils than soybean growth. Compared with LDPP-MP, enzymes in soils change LDPE-MP more easily when MP sizes are small with a rough surface. MP change is also influenced by the SA and the dose effect of MP.

## Data Availability Statement

The original contributions presented in the study are included in the article/Supplementary Material, further inquiries can be directed to the corresponding author.

## Author Contributions

JW and SZ contributed to the conception and design of the study, manuscript revision, read, and approved the submitted version. JW wrote the first draft of the manuscript. SZ contributed to supervision and funding acquisition. MA was responsible for the language polishing of the article. All authors contributed to the experiment and data collection.

## Conflict of Interest

The authors declare that the research was conducted in the absence of any commercial or financial relationships that could be construed as a potential conflict of interest.

## Publisher’s Note

All claims expressed in this article are solely those of the authors and do not necessarily represent those of their affiliated organizations, or those of the publisher, the editors and the reviewers. Any product that may be evaluated in this article, or claim that may be made by its manufacturer, is not guaranteed or endorsed by the publisher.
