# Supplementary material for: Crops Change the Morphology, Abundance, and Mass of Microplastics in Mollisols of Northeast China
Source: Front Microbiol. 2022 Apr 4;13:733804. doi: 10.3389/fmicb.2022.733804 (PMC9014289; doi:10.3389/fmicb.2022.733804)
Supplement: Supplementary file 1 [file Data_Sheet_2.DOCX]

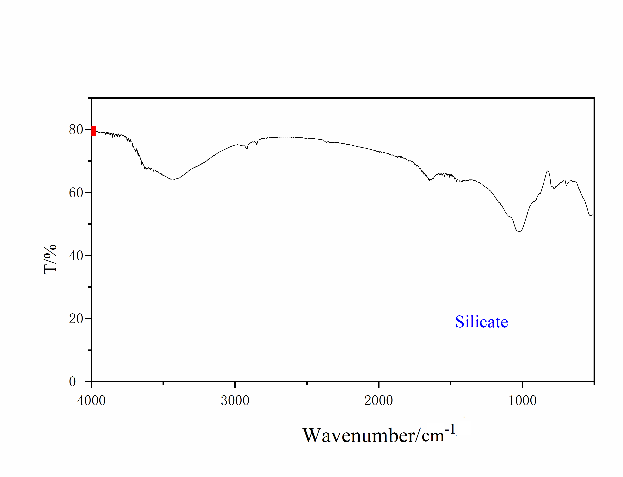

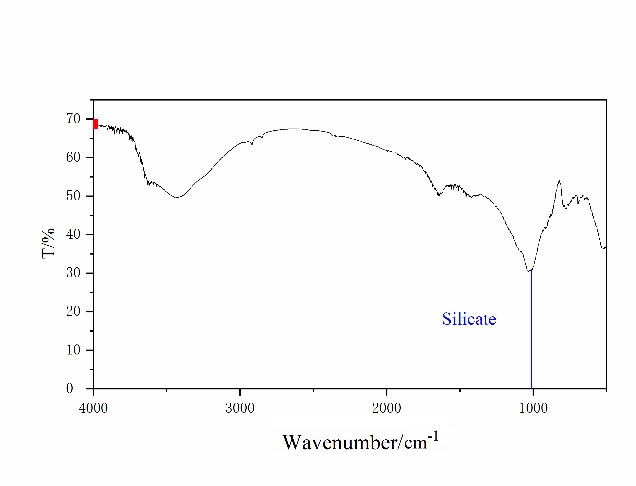

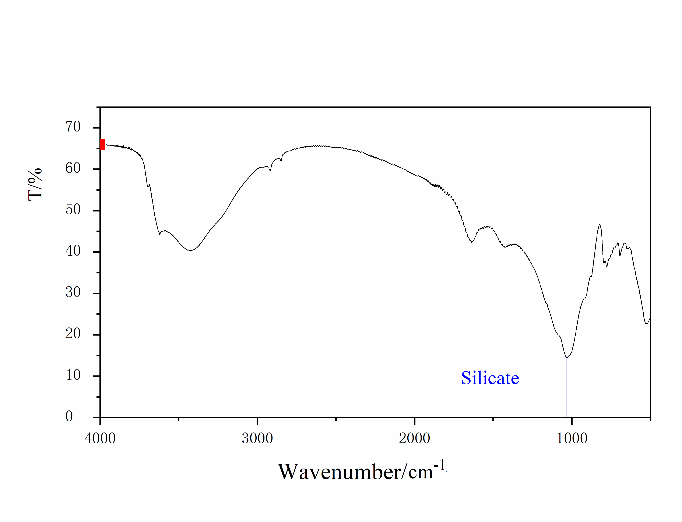


**Figure S1** Soils used for lab incubation were examined by the Fourier infrared spectra (3 times), and no plastics pollution was found.

**
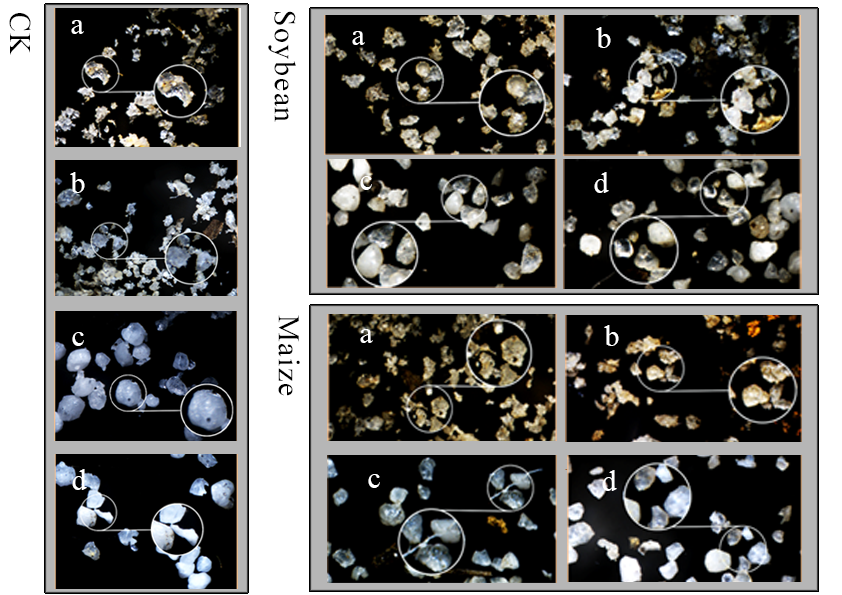
Figure S2**. Surface morphology of low-density polyethylene (LDPE) and low-density polypropylene (LDPP) in treatments of crop plantation (Maize) and straw addition after incubation. Sub-figure a was only PE addition. Sub-figure b was PE with straw amendment. Sub-figure c was only PP addition. Sub-figure d was PP with straw amendment. Images were taken by the stereomicroscope combined with microinfrared spectrum. CK was the control treatment without crop plantation. Here, only treatments of 1% MP were shown.

CK JPP


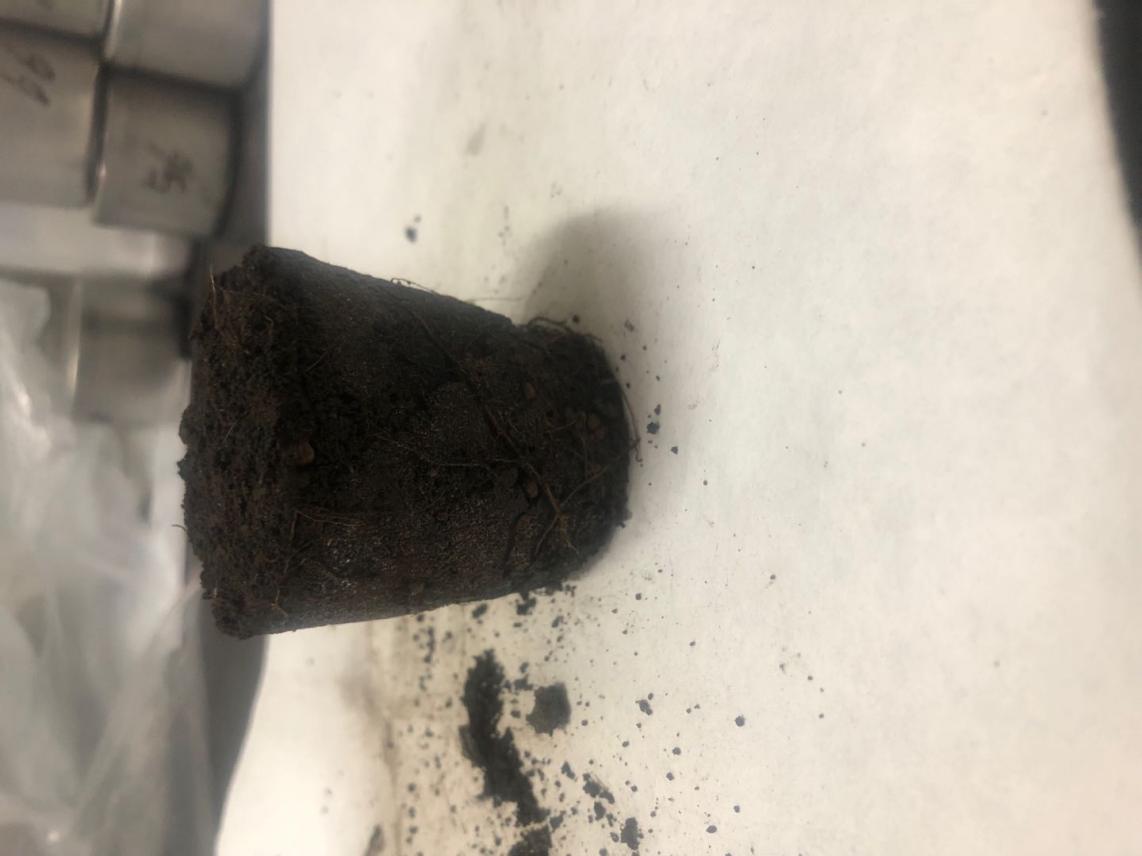

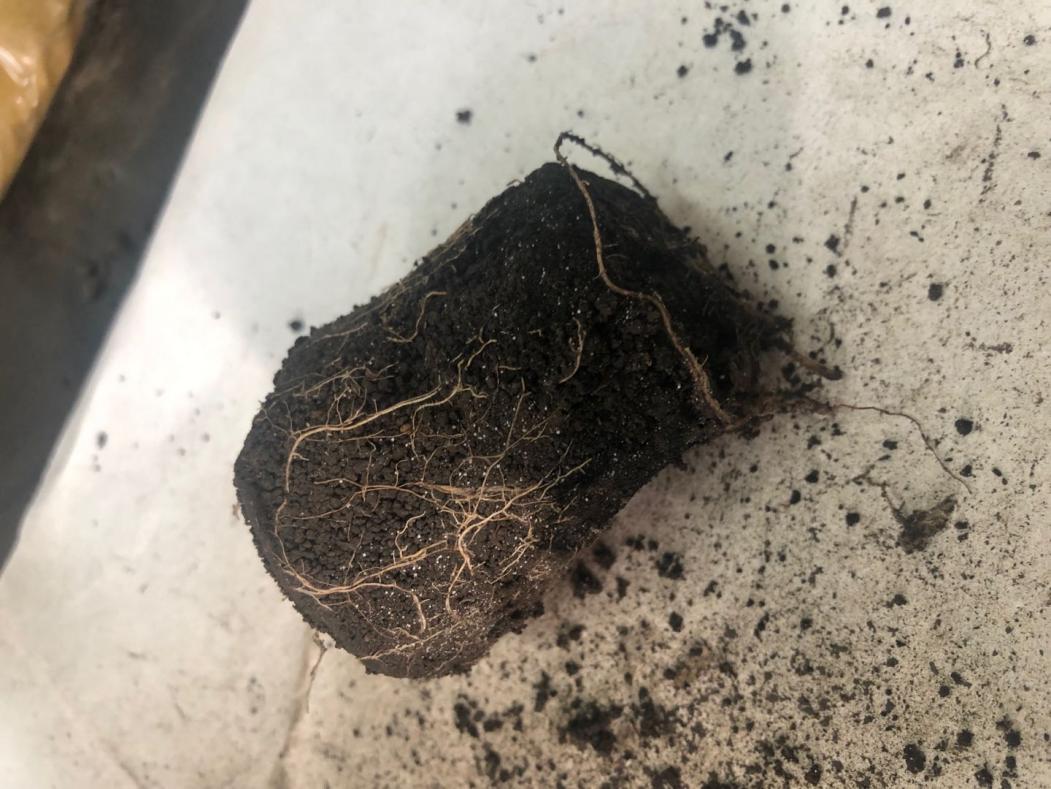


**b**

**a**


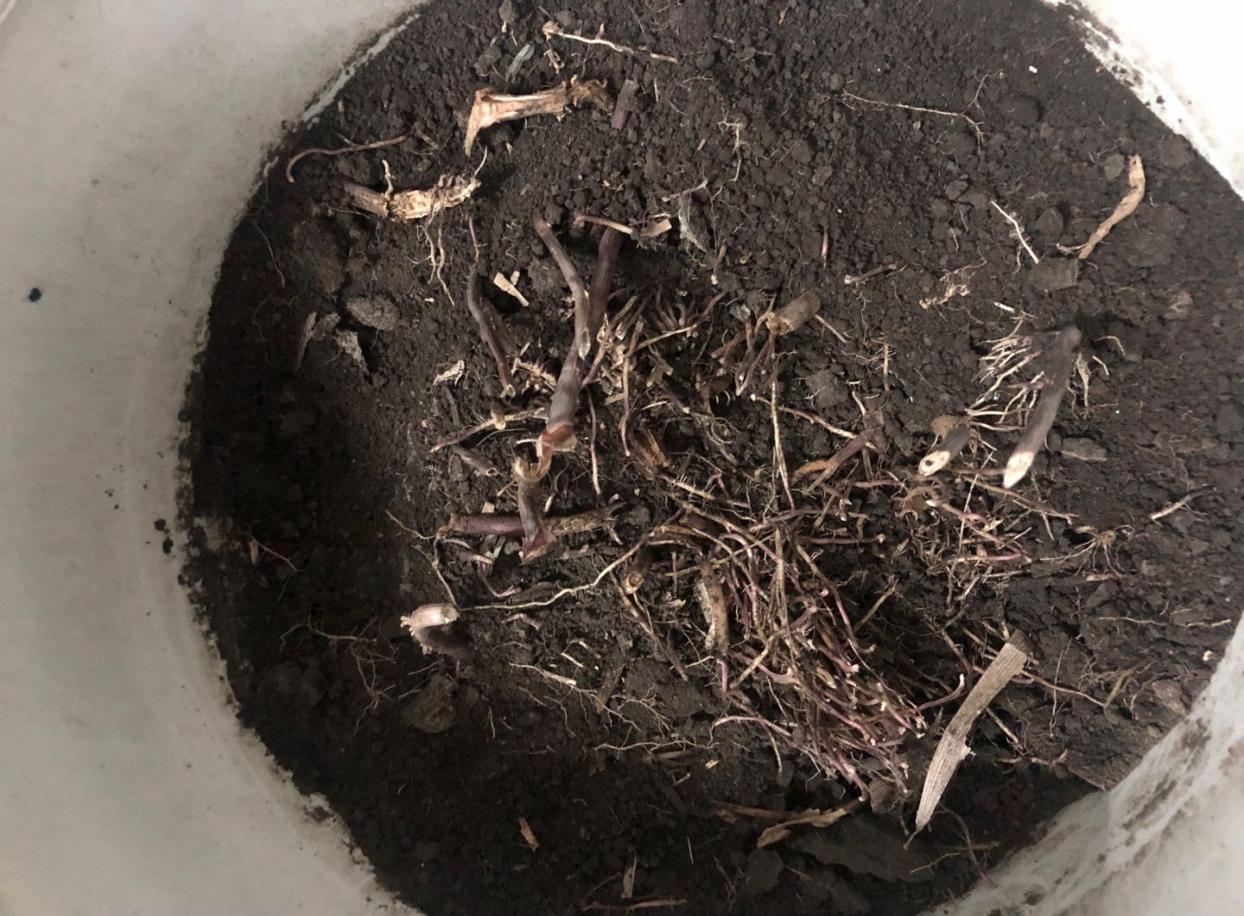


**c**

**Figure S3**. Photos of root distribution in cups differed between crop types. Sub-figure a was soybean (1% PP), sub-figure b was maize (1% PP), sub-figure c was maize (bucket)
